# Extracellular Vesicles from Amnion-Derived Mesenchymal Stem Cells Ameliorate Hepatic Inflammation and Fibrosis in Rats

**DOI:** 10.1155/2018/3212643

**Published:** 2018-12-24

**Authors:** Masatsugu Ohara, Shunsuke Ohnishi, Hidetaka Hosono, Koji Yamamoto, Kohei Yuyama, Hideki Nakamura, Qingjie Fu, Osamu Maehara, Goki Suda, Naoya Sakamoto

**Affiliations:** ^1^Department of Gastroenterology and Hepatology, Hokkaido University Graduate School of Medicine, Sapporo 0608638, Japan; ^2^Laboratory of Biomembrane and Biofunctional Chemistry, Graduate School of Advanced Life Science, Hokkaido University, Sapporo 0600810, Japan; ^3^Central Research Institute, Hokkaido University Graduate School of Medicine, Graduate School of Dental Medicine, Sapporo 0608638, Japan; ^4^Department of Pathophysiology and Therapeutics, Faculty of Pharmaceutical Sciences, Hokkaido University, Sapporo 0600812, Japan

## Abstract

**Background:**

There are no approved drug treatments for liver fibrosis and nonalcoholic steatohepatitis (NASH), an advanced stage of fibrosis which has rapidly become a major cause of cirrhosis. Therefore, development of anti-inflammatory and antifibrotic therapies is desired. Mesenchymal stem cell- (MSC-) based therapy, which has been extensively investigated in regenerative medicine for various organs, can reportedly achieve therapeutic effect in NASH via paracrine action. Extracellular vesicles (EVs) encompass a variety of vesicles released by cells that fulfill functions similar to those of MSCs. We herein investigated the therapeutic effects of EVs from amnion-derived MSCs (AMSCs) in rats with NASH and liver fibrosis.

**Methods:**

NASH was induced by a 4-week high-fat diet (HFD), and liver fibrosis was induced by intraperitoneal injection of 2 mL/kg 50% carbon tetrachloride (CCl_4_) twice a week for six weeks. AMSC-EVs were intravenously injected at weeks 3 and 4 in rats with NASH (15 *μ*g/kg) and at week 3 in rats with liver fibrosis (20 *μ*g/kg). The extent of inflammation and fibrosis was evaluated with quantitative reverse transcription polymerase chain reaction and immunohistochemistry. The effect of AMSC-EVs on inflammatory and fibrogenic response was investigated *in vitro*.

**Results:**

AMSC-EVs significantly decreased the number of Kupffer cells (KCs) in the liver of rats with NASH and the mRNA expression levels of inflammatory cytokines such as tumor necrosis factor- (*Tnf-*) *α*, interleukin- (*Il-*) *1β* and *Il-6*, and transforming growth factor- (*Tgf-*) *β*. Furthermore, AMSC-EVs significantly decreased fiber accumulation, KC number, and hepatic stellate cell (HSC) activation in rats with liver fibrosis. *In vitro*, AMSC-EVs significantly inhibited KC and HSC activation and suppressed the lipopolysaccharide (LPS)/toll-like receptor 4 (TLR4) signaling pathway.

**Conclusions:**

AMSC-EVs ameliorated inflammation and fibrogenesis in a rat model of NASH and liver fibrosis, potentially by attenuating HSC and KC activation. AMSC-EV administration should be considered as a new therapeutic strategy for chronic liver disease.

## 1. Introduction

Chronic tissue injury leads to sustained scarring response that gradually disrupts normal cellular function, eventually leading to failure in multiple epithelial organs such as the lung, kidney, and liver [1]. Nonalcoholic fatty liver disease (NAFLD) is the most common cause of chronic liver disease worldwide, and nonalcoholic steatohepatitis (NASH) is rapidly becoming one of the main causes of cirrhosis, hepatocellular carcinoma, and indications for liver transplantation; however, there are no approved drug treatments for NAFLD and NASH [2, 3]. Cirrhosis, the terminal stage of progressive liver fibrosis, results in portal hypertension, hepatic encephalopathy, and liver failure [1, 4]. The only available therapeutic approaches are removal of the injurious stimuli and liver transplantation, which is limited by organ shortage, high expense, and requirement of lifelong immunosuppressive therapy [5, 6]. Therefore, the development of anti-inflammatory and antifibrotic therapies is a major unmet clinical need for liver fibrosis and NASH.

Mesenchymal stem cells (MSCs) are multipotent cells capable of differentiating into a variety of specialized cells such as osteoblasts, chondrocytes, and adipocytes [7]. MSC-based therapy has been extensively investigated in regenerative medicine for various organs [8, 9]. We previously demonstrated that systemic administration of amnion-derived MSCs (AMSCs) in rats led to the improvement of severe colitis [10, 11], radiation proctitis [12], pancreatitis [13], and liver fibrosis [14], potentially through secretory factors released from transplanted AMSCs. Previous studies showed that MSCs can achieve therapeutic effect via paracrine action and direct differentiation *in vivo* [15, 16]. However, most of the transplanted cells are estimated not to actually reach target tissue, with most of the cells trapped in the lung, liver, and spleen [17]. Furthermore, we recently demonstrated that administration of conditioned medium from AMSC culture led to the improvement of severe colitis in rats [10] and esophageal and rectal strictures after large endoscopic submucosal dissection in pigs [18, 19].

Extracellular vesicles (EVs) comprise a variety of vesicles that are released into the extracellular environment by cells [20], which are categorized as exosomes (EXs, 30–120 nm), microvesicles (MVs, 50 nm–1 *μ*m), and apoptotic bodies based on their biogenesis pathways [21]. Given that no specific EX and MV markers have been identified yet, the International Society for Extracellular Vesicles agreed to consider all experimentally obtained vesicles as EVs [22]. MSC-derived EVs were reported to exert functions similar to those of MSCs including induction of cell proliferation as well as anti-inflammatory, immunomodulatory, antifibrotic, and antiapoptotic effects [23].

Thus, the aim of this study was to examine the effect of EVs obtained from human AMSCs on high-fat diet- (HFD-) induced NASH and carbon tetrachloride- (CCl_4_-) induced liver fibrosis in rats and to investigate the underlying mechanisms.

## 2. Materials and Methods

### 2.1. Isolation and Expansion of AMSCs and Normal Skin Fibroblasts

For the isolation and expansion of AMSCs and normal skin fibroblasts (NFs), after approval obtained from the Medical Ethics Committee of Hokkaido University Graduate School of Medicine in Sapporo, Japan, written informed consent was obtained from all pregnant women and patients. AMSCs were isolated as described previously [18] and we confirmed that AMSCs were multipotent and expressed MSC markers including CD44, CD73, CD90, and CD105 but not hematopoietic markers including CD11b, CD19, CD34, CD45, and human leukocyte antigen-DR [11, 14, 18]. NFs were isolated as described previously [24]. AMSCs and NFs were cultured with minimal essential medium- (MEM-) *α* (Life Technologies, Carlsbad, CA, USA) supplemented with 10% fetal bovine serum (Life Technologies), 100 U/mL penicillin, and 100 *μ*g/mL streptomycin (Wako Pure Chemical Industries, Osaka, Japan).

### 2.2. Isolation of EVs from AMSCs and NFs

EVs were isolated and purified from AMSCs and NFs to obtain AMSC-EVs and NF-EVs, respectively, as described previously with minor modifications [25, 26]. Cell cultures were maintained at 37°C in a humidified atmosphere of 95% air and 5% CO_2_. When cultured cells reached 80%–90% confluence, the culture medium was replaced by serum-free medium after washing with phosphate-buffered saline (PBS, Life Technologies) three times, and the cells were cultured for an additional 48 hours. The conditioned medium was collected and centrifuged at 2500 g for 5 minutes at 4°C to remove cellular debris. Next, the supernatants were filtered using a 0.22 *μ*m filter (Steritop, Merck Millipore, Billerica, MA, USA). The filtrates were ultracentrifuged at 100,000 g for 70 minutes at 4°C. The pellets were diluted with PBS and ultracentrifuged at 100,000 *g* for 70 minutes at 4°C. Finally, the resulting EV pellets were resuspended and stored at −80°C until use.

### 2.3. Identification of AMSC-EVs and NF-EVs

Size distribution of the EVs was determined using the qNano system (Izon Science, Christchurch, New Zealand) according to the manufacturer's protocol. Protein content of the concentrated EVs was measured using the Qubit protein assay kit (Life Technologies) and Qubit 2.0 (Life Technologies). Expression of the typical EV marker CD81 was verified by western blotting using an antibody (1 : 200) from HansaBioMed Life Sciences (Tallinn, Estonia). AMSC-EVs were also identified by scanning electron microscopy (SEM).

### 2.4. SEM

EVs were fixed in 2% glutaraldehyde for at least four hours and processed for SEM. Briefly, samples were dehydrated in a graded ethanol series, treated twice with isoamyl acetate, and dried to a critical point with a Hitachi HCP-2 dryer (Hitachi, Tokyo, Japan), followed by optional platinum-palladium sputter coating in a Hitachi E-1030 device (Hitachi). Specimens were photographed using a Hitachi S-4500 SEM (Hitachi) fitted with a digital image capturing system.

### 2.5. Animals

The experimental protocol for this study was approved by the Animal Care Unit and Use Committee of Hokkaido University. Six-week-old male Sprague-Dawley rats were procured from Japan SLC (Hamamatsu, Japan), and three rats were housed per cage in a temperature-controlled room (24°C) on a 12-hour/12-hour light/dark cycle. All rats had *ad libitum* access to water.

### 2.6. NASH Model and AMSC-EV Treatment

**Figure 1:**
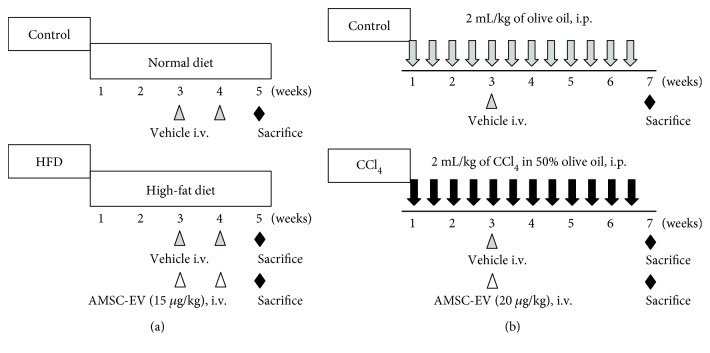
Protocol for animal experiments. (a) Experimental protocol for HFD-induced NASH model. Rats received HFD for four weeks. AMSC-EVs (15 *μ*g/kg) were infused intravenously (i.v.) at weeks 3 and 4. All rats were sacrificed at week 5. (b) Experimental protocol for CCl_4_-induced liver fibrosis. Rats received intraperitoneal (i.p.) injection 2 mL/kg CCl_4_ in 50% olive oil twice a week for six weeks. AMSC-EVs (20 *μ*g/kg) were infused intravenously at week 3. All rats were sacrificed at week 7. HFD: high-fat diet; NASH: nonalcoholic steatohepatitis; AMSC: amnion-derived mesenchymal stem cell; EV: extracellular vesicle; CCl_4_: carbon tetrachloride.

The NASH model was generated by feeding rats (*n* = 22) on a HFD (Oriental Yeast, Tokyo, Japan) for four weeks, whereas rats in the control group (*n* = 6) were given a normal diet (Oriental Yeast). AMSC-EVs (15 *μ*g/kg) suspended in 200 *μ*L PBS were intravenously injected into the HFD rats (HFD + AMSC-EV group, *n* = 11) through the penile vein at weeks 3 and 4. Conversely, 200 *μ*L PBS was injected via the same route in control rats and those treated with HFD (HFD group, *n* = 11). All rats were sacrificed at five weeks after HFD initiation (Figure 1(a)).

### 2.7. Liver Fibrosis Model and AMSC-EV Treatment

Liver fibrosis was induced by intraperitoneal injection of 2 mL/kg 50% CCl_4_ (Wako Pure Chemical Industries) resuspended in olive oil twice a week for six weeks. In the control group (*n* = 6), rats were injected with olive oil alone. AMSC-EVs (20 *μ*g/kg) suspended in 200 *μ*L PBS were intravenously injected through the penile vein at week 3 after the start of CCl_4_ treatment (CCl_4_ + AMSC-EV group, *n* = 15). Conversely, 200 *μ*L PBS was injected to the control rats and those treated with CCl_4_ (CCl_4_ group, *n* = 15). All rats were sacrificed at week 7 after CCl_4_ initiation (Figure 1(b)).

### 2.8. Histological Examination

Left lobe of the liver was removed, fixed in 40 g/L formaldehyde in saline, embedded in paraffin, and cut into 5 *μ*m sections. Tissue sections were stained with Masson's trichrome. Ten random fields on a section from each rat were photographed (×100), and blue-stained areas were measured from the entire cross-sectional area of the liver specimen and expressed as percentages using the WinROOF digital image analyzer (Mitani, Fukui, Japan). We followed the methods of Ohara et al. [27].

### 2.9. Immunohistochemical Examination

To assess the activation of hepatic stellate cells (HSCs), tissue sections were stained with an anti-rat *α*-smooth muscle actin (SMA) antibody (1 : 800, Thermo Scientific, Waltham, MA, USA) for 30 minutes at room temperature. To assess the number of Kupffer cells (KCs), tissue sections were stained with an anti-rat CD68 monoclonal antibody (1 : 50; AbD Serotec, Kidlington, United Kingdom) for 40 minutes at room temperature. Ten random fields on a section from each rat were photographed, and stained areas were measured as percentage of the entire liver cross-sectional area.

### 2.10. RNA Isolation and Quantitative Reverse Transcription Polymerase Chain Reaction

Total RNA from rat liver or cultured cells was extracted using the RNeasy Mini kit (Qiagen, Hilden, Germany), and 1 *μ*g total RNA was reverse-transcribed into cDNA using PrimeScript™ RT reagent kit (Takara Bio, Kusatsu, Japan). Polymerase chain reaction (PCR) amplification was performed using a 25 *μ*L reaction mixture that contained 1 *μ*L cDNA and 12.5 *μ*L Platinum SYBR Green PCR mix (Invitrogen, Carlsbad, CA, USA). *β*-Actin and cytoplasmic 18S small subunit ribosomal RNA (rRNA) messenger RNA that were amplified from the same samples served as internal controls. After initial denaturation at 95°C for 2 minutes, a two-step cycle protocol was used: denaturation at 95°C for 15 seconds and annealing and extension at 60°C for 1 minute, for 40 cycles in a 7700 Sequence Detector (Applied Biosystems, Foster City, CA, USA). Gene expression levels were determined using the comparative threshold cycle (ΔΔCt) method with *β*-actin and 18S rRNA used as endogenous controls. Data were analyzed with the Sequence Detection Systems software (Applied Biosystems). Primer sequences are shown in Table 1.

### 2.11. In Vitro Experiments Using Rat HSCs and KCs

Isolation of HSCs and KCs from rat liver was performed as described previously with modification [28]. Briefly, rat liver was treated with *in situ* pronase/collagenase perfusion and subsequently digested *in vitro*. Next, separation of HSCs and KCs from other hepatic cell populations was achieved by a density gradient-based protocol using two different concentrations of Ficoll PM400 (GE Healthcare, Chicago, IL, USA).

### 2.12. Western Blotting

To investigate the phosphorylation of inhibitor of kappa (I*κ*)B-*α* and p65, HEK293 human embryonic kidney cells provided by the RIKEN BioResource Center (Tsukuba, Japan) or a cell line derived from HEK293 cells which were stably transfected with human toll-like receptor (TLR) 4a, MD2, and CD14 (293/hTLR4A-MD2-CD14; InvivoGen, San Diego, CA, USA) was plated into 6-well plates (Corning, Corning, NY, USA) at a density of 2 × 10^6^ cells/well and cultured in complete medium. The next day, the culture medium was changed to a medium containing AMSC-EVs or PBS, and cells were incubated for 60 minutes, followed by treatment with 10 ng/mL lipopolysaccharide (LPS, Sigma-Aldrich, St. Louis, MO, USA) for two hours. Next, cells were washed with ice-cold PBS, and cell lysates were prepared using radio-immunoprecipitation assay buffer containing 50 mM Tris-HCl (pH 8.0), 150 mM NaCl, 0.5% (w/v) sodium deoxycholate, 0.1% (w/v) sodium dodecyl sulfate (SDS), 1.0% (w/v) NP-40 substitute, and protease/phosphatase inhibitor cocktail (Cell Signaling Technology, Beverly, MA, USA). Equal amounts of cellular protein extracts were diluted in 4× Laemmli sample buffer (Bio-Rad, Hercules, CA, USA). The samples were heated at 95°C for 5 minutes, separated using SDS-polyacrylamide gel electrophoresis (Bio-Rad), and transferred to Immobilon-P polyvinylidene difluoride membranes (Merck Millipore). The membranes were incubated in Tris-buffered saline with 0.05% Tween 20 (Wako Pure Chemical Industries) and 5% PhosphoBLOCKER blocking reagent (Cell Biolabs, San Diego, CA, USA) at room temperature for 60 minutes. The membranes were probed with primary antibodies for phospho-I*κ*B-*α* (1 : 2000), I*κ*B-*α* (1 : 2000), phospho-p65 (1 : 2000), and nuclear factor kappa B (NF-*κ*B, 1 : 2000), all from Cell Signaling Technology, and bound antibodies were detected with peroxidase-conjugated AffiniPure goat anti-mouse IgG (H + L) (1 : 10,000; Jackson ImmunoResearch, West Grove, PA, USA) or peroxidase-conjugated AffiniPure goat anti-rabbit IgG (H + L) (1 : 10,000; Jackson ImmunoResearch) antibodies. To investigate the expression of EV marker CD81, EVs were diluted in 4× Laemmli sample buffer (Bio-Rad). The samples were heated at 95°C for 5 minutes, separated using SDS-polyacrylamide gel electrophoresis (Bio-Rad), and transferred to Immobilon-P polyvinylidene difluoride membranes. The membranes were blocked with Blocking One (Nacalai Tesque, Kyoto, Japan) at room temperature for 60 minutes. The membranes were probed with primary antibody for CD81 (1 : 200, HansaBioMed Life Sciences, Tallinn, Estonia) and detected with peroxidase-conjugated AffiniPure goat anti-rabbit IgG (H + L) (1 : 2000). The membranes were visualized using ECL Prime detection reagent (GE Healthcare), and the blots were analyzed using ImageQuant LAS-4000 (Fujifilm, Tokyo, Japan).

### 2.13. Transient Transfection and Reporter Gene Assay

HEK293 and 293/hTLR4-MD2-CD14 cells (1.25 × 10^5^ cells/well) were plated into 24-well plates (Corning) containing 500 *μ*L culture medium. After incubation for 24 hours at 37°C, the cells were transfected with 25 ng luciferase plasmid DNA, 25 ng Renilla pGL4.74 (hRluc/TK) vector (Promega, Madison, WI, USA) as an internal control, 500 ng plasmid DNA containing five copies of an NF-*κ*B response element that drove the transcription of the luciferase reporter gene (pGL4.32 [luc2P/NF-*κ*B RE/Hygro]; Promega), and/or a tumor necrosis factor (TNF) receptor-associated factor 6 (TRAF6) plasmid using Lipofectamine® LTX (Life Technologies). TRAF6 plasmid was a gift by Dr. Koji Nakagawa (Hokkaido University). After 24 hours of incubation at 37°C, the cells were treated with 10 ng/mL LPS for six hours, and the reporter gene assay was performed using the Dual Luciferase® reporter assay system (Promega). Luminescence intensity was measured using the GloMax®-Multi Detection System (Promega) according to the manufacturer's instructions. Transcriptional activity was normalized to the Renilla luciferase activity. All experiments were performed in triplicate.

### 2.14. Statistical Analysis

Data were expressed as means ± standard deviation (SD). Parameters among the groups were compared by one-way analysis of variance followed by Tukey's test. Intergroup comparisons were achieved using unpaired Student's *t*-test. All differences were considered as significant at a *p* < 0.05. All analyses were performed using GraphPad Prism version 7 (GraphPad, San Diego, CA, USA).

## 3. Results

### 3.1. Characterization of AMSC-EVs and NF-EVs

**Figure 2:**
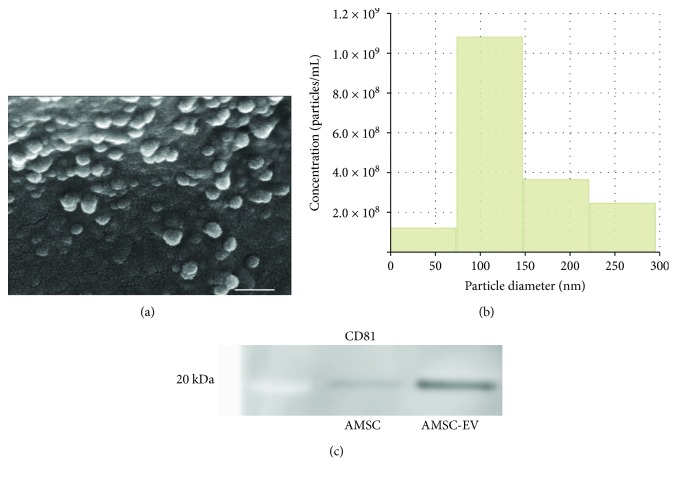
Characterization of AMSC-EVs. (a) Scanning electron micrograph of fixed and dehydrated EVs. (b) Size distribution of the particles measured with the qNano system. (c) Western blot analysis with anti-CD81 antibody. Scale bar, 100 nm. AMSC: amnion-derived mesenchymal stem cell; EV: extracellular vesicle.

First, we examined AMSC-EVs by SEM (Figure 2(a)), and qNano system demonstrated that the diameter distribution of AMSC-EVs ranged from 50 to 150 nm, with a single peak at approximately 100 nm (Figure 2(b)). Additionally, we confirmed the expression of the EV marker CD81 by western blotting of AMSC-EVs (Figure 2(c)). The size distribution of NF-EVs ranged from 80 to 110 nm, with a single peak at approximately 90 nm (Supplementary Figure 1A), and NF-EVs also expressed the EV marker CD81 (Supplementary Figure 1B).

### 3.2. AMSC-EVs Alleviate Inflammatory Response in a Rat Model of NASH

**Figure 3:**
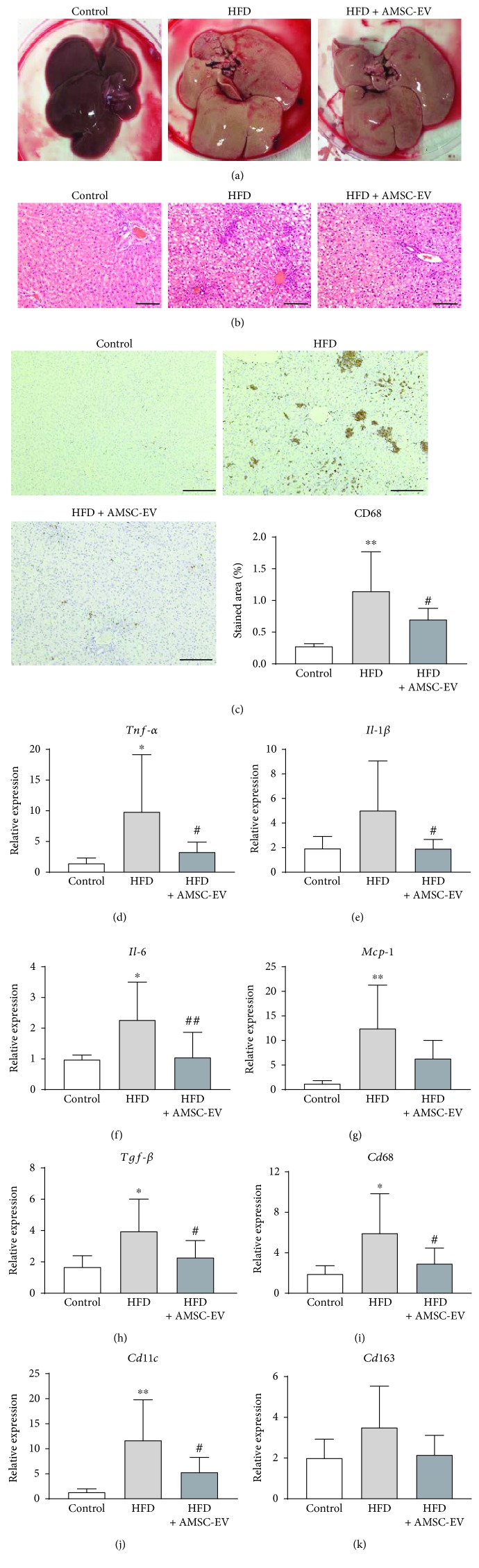
Effect of AMSC-EVs in rats with HFD-induced steatohepatitis. (a) Gross appearance of the dissected liver. (b) Hematoxylin and eosin staining. (c) Expression of CD68. (d–k) Quantitative reverse transcription polymerase reaction for *Tnf-α*, *Il-1β*, *Il-6*, *Mcp-1*, *Tgf-β*, *Cd68*, *Cd11c*, and *Cd163*, respectively. Data are presented as means ± standard deviation. ^∗^ *p* < 0.05, ^∗∗^ *p* < 0.01 versus control, ^#^ *p* < 0.05, ^##^ *p* < 0.01 versus HFD. Scale bar, 200 *μ*m. AMSC: amnion-derived mesenchymal stem cell; EV: extracellular vesicle; HFD: high-fat diet; *Tnf-α*: tumor necrosis factor-*α*; *Il*: interleukin; *Mcp-1*: monocyte chemoattractant protein-1; *Tgf-β*: transforming growth factor-*β*.

We next evaluated the role of AMSC-EVs in a rat model of NASH. Comparison of the liver specimens from six rats in the control group with eleven rats in each of the HFD and HFD + AMSC-EV groups revealed that the livers in the HFD and HFD + AMSC-EV groups were enlarged and exhibited a fatty appearance (Figure 3(a)). Hematoxylin and eosin staining demonstrated the accumulation of lipid droplets and infiltration of mononuclear cells in the livers of HFD and HFD + AMSC-EV groups (Figure 3(b)). Immunohistological examination demonstrated that the number of KCs, determined by the expression of the KC marker CD68, was significantly increased in the HFD group; however, AMSC-EVs significantly decreased the number of KCs (Figure 3(c)). On the other hand, the expression of CD163, a marker for M2 macrophages, was not significantly different between the HFD and HFD + AMSC-EV groups (Supplementary Figure 2). Quantitative reverse transcription PCR demonstrated that HFD significantly increased the expression levels of *Tnf-α*, interleukin- (*Il-) 6*, and monocyte chemoattractant protein- (*Mcp-) 1* (Figures 3(d), 3(f), and 3(g), respectively), and the expressions of *Tnf-α*, *Il-1β*, *and Il-6* were significantly reduced by AMSC-EVs (Figure 3(d)–3(f), respectively). Furthermore, the expression level of transforming growth factor- (*Tgf-) β* was significantly decreased by AMSC-EVs (Figure 3(h)). In the HFD group, mRNA expression levels of macrophage markers such as *Cd68* and *Cd11c*, a marker that identifies M1 macrophages, were significantly increased, which were significantly decreased in animals treated with AMSC-EVs (Figures 3(i) and 3(j), respectively). The expression level of the M2 macrophage marker *Cd163* was not significantly different among the three treatment groups (Figure 3(k)).

### 3.3. AMSC-EVs Suppress Fibrosis in a Rat Model of Liver Fibrosis

**Figure 4:**
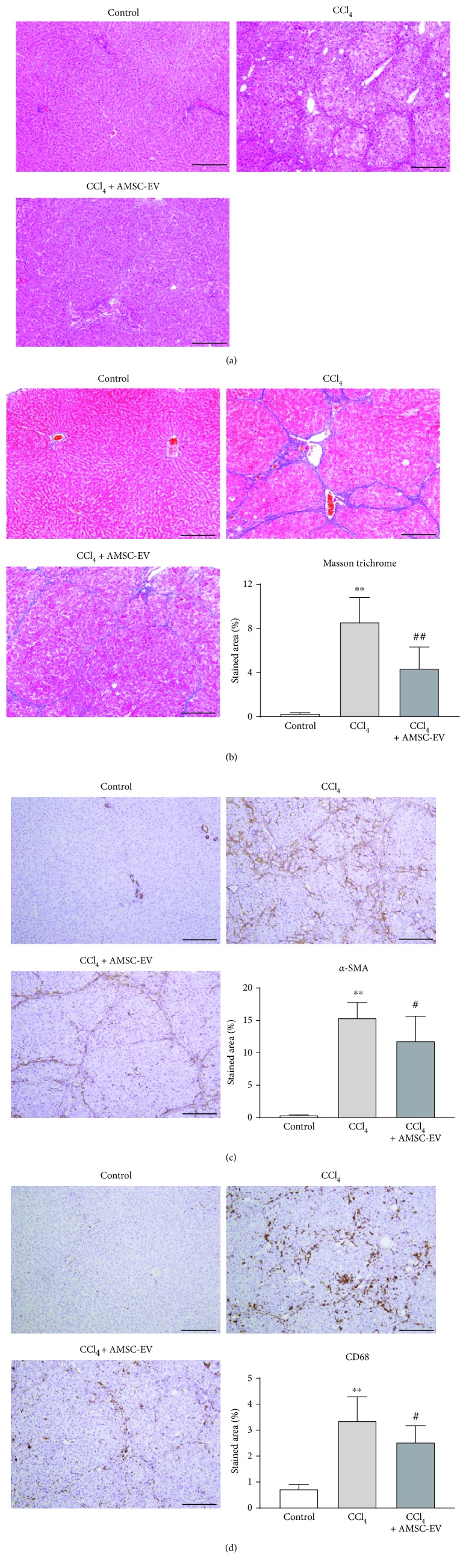
Effect of AMSC-EVs in rats with CCl_4_-induced liver fibrosis. (a) Hematoxylin and eosin staining. (b) Masson trichrome staining. (c) Expression of *α*-smooth muscle action (SMA). (d) Expression of CD68. Data are presented as means ± standard deviation. ^∗∗^ *p* < 0.01 versus control, ^#^ *p* < 0.05, ^##^ *p* < 0.01 versus CCl_4_. Scale bar, 200 *μ*m. AMSC: amnion-derived mesenchymal stem cell; EV: extracellular vesicle; CCl_4_: carbon tetrachloride.

We next assessed the effect of AMSC-EVs in a rat model of liver fibrosis. Fifteen rats in each of the CCl_4_ and CCl_4_ + AMSC-EV groups were compared with six rats in the control group. Whereas five rats died at weeks 6 and 7 in the CCl_4_ group, four rats died at weeks 6 and 7 in the CCl_4_ + AMSC-EV group. Therefore, 6, 10, and 11 rats from the control, CCl_4_, and CCl_4_ + AMSC-EV groups, respectively, were analyzed. Hematoxylin and eosin staining demonstrated that the thick fibrotic septa and pseudolobule formation were more extensive in the CCl_4_ group compared with the control and CCl_4_ + AMSC-EV groups (Figure 4(a)). Severe fibrosis was observed in the CCl_4_ group; however, fiber accumulation was significantly attenuated by AMSC-EV treatment at week 7 (Figure 4(b)). The expression of *α*-SMA, a marker for HSC activation, was significantly increased in the CCl_4_ group, which was significantly attenuated by AMSC-EV treatment (Figure 4(c)). The expression of CD68 was significantly increased in the CCl_4_ group; however, AMSC-EV treatment decreased the number of CD68-positive KCs (Figure 4(d)).

### 3.4. AMSC-EVs Suppress LPS-Induced Activation of KCs and HSCs In Vitro

**Figure 5:**
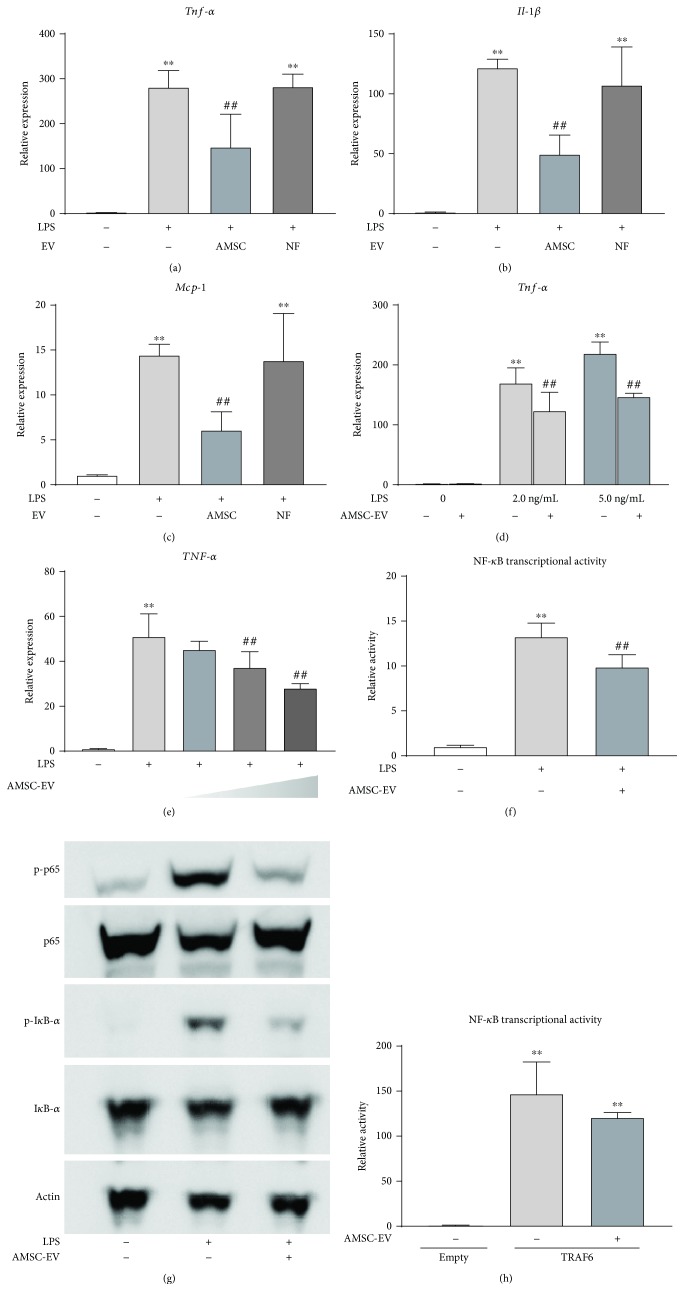
Effect of AMSC-EVs and NF-EVs on inflammatory response in cultured KCs and HSCs. (a–c) KCs were treated with 2.5 ng/mL LPS for three hours after pretreatment with AMSC-EVs or NF-EVs (0.5 *μ*g/well in 12-well plate) for 30 minutes. Total RNA was isolated after LPS administration, and the expressions of *Tnf-α*, *Il-1β*, and *Mcp-1* were determined by qRT-PCR. (d) AMSC-EVs were added to cultured HSCs together with 2 or 5 ng/mL LPS. Total RNA was isolated three hours after LPS treatment, and *Tnf-α* expression was determined by qRT-PCR. (e) AMSC-EVs were added to cultured 293/hTLR4A-MD2-CD14 cells together with 5 ng/mL LPS. Total RNA was isolated three hours after LPS treatment, and *Tnf-α* expression was determined by qRT-PCR. (f) Effect of AMSC-EVs on transcriptional activity of NF-*κ*B in 293/hTLR4A-MD2-CD14 cells treated with LPS (10 ng/mL) for six hours. (g) 293h/TLR4A-MD-2-CD14 cells were treated with LPS (10 ng/mL) for two hours after pretreatment with AMSC-EVs for one hour, and the expression of phosphorylation of I*κ*B-*α* and p65 was determined by western blotting. (h) Effect of AMSC-EVs on transcriptional activity of NF-*κ*B in HEK293 cells overexpressing TNF receptor-associated factor 6 (TRAF6). Data are presented as means ± standard deviation. ^∗∗^ *p* < 0.01 versus control, ^##^ *p* < 0.01 versus LPS. AMSC: amnion-derived mesenchymal stem cell; EV: extracellular vesicle; NF: normal skin fibroblast; KC: Kupffer cell; HSC: hepatic stellate cell; LPS: lipopolysaccharide; *Tnf-α*: tumor necrosis factor-*α*; *Il*: interleukin; *Mcp-1*: monocyte chemoattractant protein-1; qRT-PCR: quantitative reverse transcription polymerase chain reaction; NF-*κ*B: nuclear factor kappa B; I*κ*B: inhibitor kappa B.

To elucidate the mechanisms underlying these *in vivo* results, we next examined the effect of AMSC-EVs on inflammatory response in cultured KCs and HSCs. Treatment with LPS markedly upregulated the expression levels of *Tnf-α*, *Il-1β*, and *Mcp-1* in KCs, which were significantly attenuated by AMSC-EV treatment (Figure 5(a)–5(c), respectively). Conversely, NF-EVs did not inhibit the expression levels of these cytokines. In addition, in HSCs, treatment with LPS significantly upregulated the expression of *Tnf-α*, which was suppressed by AMSC-EVs (Figure 5(d)).

We next examined whether AMSC-EVs inhibited LPS-induced NF-*κ*B activation in 293/hTLR4A-MD2-CD14 and HEK293 cells. We confirmed that AMSC-EVs dose-dependently suppressed the expression of *TNF-α* (Figure 5(e)). In addition, the increase in LPS-induced NF-*κ*B transcriptional activity was significantly suppressed by AMSC-EVs (Figure 5(f)). Furthermore, LPS-induced phosphorylation of I*κ*B-*α* and p65 was inhibited by AMSC-EVs (Figure 5(g)). Whereas NF-*κ*B transcriptional activity was upregulated by overexpression of TRAF6, AMSC-EVs did not downregulate the NF-*κ*B transcriptional activity (Figure 5(h)), suggesting that AMSC-EVs might suppress the earlier steps of the LPS/TLR4 signaling pathway.

### 3.5. AMSC-EVs Suppress the Activation of Primary HSCs

**Figure 6:**
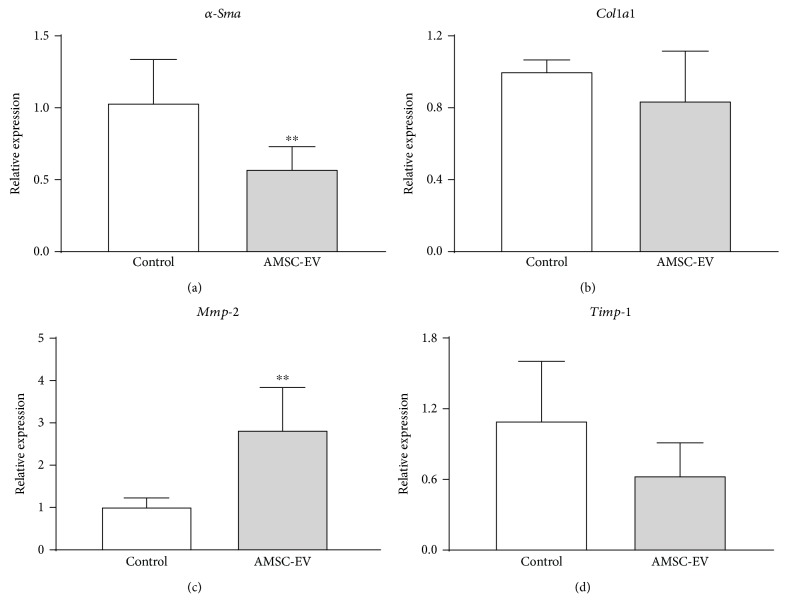
Effect of EVs obtained from AMSC cultures on activation of HSCs. Expression levels of (a) *α-Sma*, (b) *Col1a1*, (c) *Mmp-2*, and (d) *Timp-1*. Data are presented as means ± standard deviation. ^∗∗^ *p* < 0.01 versus control. AMSC: amnion-derived mesenchymal stem cell; EV: extracellular vesicle; HSC: hepatic stellate cell; *α-Sma*: *α*-smooth muscle actin; *Col1a1*: collagen1a1; *Mmp-2*: matrix metalloproteinase-2; *Timp-1*: tissue inhibitor of matrix metalloproteinases-1.

To investigate the effect of AMSC-EVs on HSC activation, HSCs were isolated from a rat liver and activated by natural culture. In these cells, AMSC-EVs significantly suppressed the expression of *α-Sma* (Figure 6(a)) and significantly increased the expression of matrix metalloproteinase-2 (*Mmp-2*, Figure 6(c)). Although the expression of collagen1a1 *(Col1a1)* was not changed (Figure 6(b)), the expression of tissue inhibitor of metalloproteinases-1 (*Timp-1*) tended to decrease in cultures treated by AMSC-EVs (Figure 6(d)).

## 4. Discussion

In the present study, we investigated the anti-inflammatory and antifibrotic effects of AMSC-EVs in rats with chronic liver disease. We found that AMSC-EV treatment improved histological findings and proinflammatory factor expressions in rats with NASH and histological changes in rats with CCl_4_-induced liver fibrosis. We further determined that AMSC-EVs suppressed the activation of cultured KCs and HSCs.

Several studies investigated the effect of MSC-derived EVs for liver injury in models of acute liver injury [9, 29–31], fulminant hepatic failure [32], CCl_4_-induced liver fibrosis [25], and thioacetamide-induced liver fibrosis [33]. EVs contain biologically active proteins, lipids, mRNAs, and miRNAs and play critical roles [34]. Among these EV components, proteins such as IL6ST/gp130, TNFRSF1A/TNFR1, and CXCL2/MIP-2 were shown to be associated with priming factors during liver regeneration [9]. However, the therapeutic effect of AMSC-EVs in NASH and liver fibrosis models has not been reported to date.

Lee et al. demonstrated that the administration of MSCs could be utilized as a clinical therapeutic tool in obesity-associated syndromes [35]. They also suggested that MSC lysates improved glucose intolerance via a paracrine effect. The concept of multiparallel hits for the development of NASH has recently been suggested [36]. In the present study, we examined inflammation in a model of NASH. In the early stages of the disease, KCs expand rapidly and secrete cytokines and chemokines such as IL-1, TNF-*α*, MCP-1, and C-C motif chemokine ligand (CCL)5 [37], reflecting their involvement in the control of inflammatory responses in NASH and their major role in the recruitment of inflammatory cells in the liver [38, 39]. CD68 is a common macrophage marker [40], and M1 macrophages commonly express CD11c [41], while M2 macrophages express CD163 and CD206 [42]. In the present study, AMSC-EVs suppressed the activation of KCs, particularly those of M1 macrophages, and downregulated the expression of inflammatory cytokines (*Tnf-α*, *Il-1β*, and *Il-6*) in rats with NASH. We also examined the role of AMSC-EVs in the context of LPS, which is recognized as one of the major factors that induce NASH [43] that was shown to directly activate KCs via TLR signaling [44]. In the LPS/TLR4 pathway, myeloid differential protein (MyD) 88 is activated, leading to TRAF6 activation and phosphorylation of p65 and I*κ*B-*α*, allowing NF-*κ*B to translocate to the nucleus [45, 46]. We previously reported that AMSCs as well as conditioned medium obtained from AMSCs exerted an anti-inflammatory effect in RAW264.7 macrophages [11]. In the present study, AMSC-EVs suppressed the expression levels of LPS-induced inflammatory cytokines in KCs and HSCs. In addition, we found that AMSC-EVs suppressed p65 and I*κ*B-*α* phosphorylation and NF-*κ*B transcriptional activity. However, AMSC-EVs did not suppress the NF-*κ*B transcriptional activity induced by overexpression of TRAF6 in HEK293 cells, suggesting that AMSC-EVs might suppress the early steps of the LPS/TLR4 signaling pathway.

Li et al. administered CCl_4_ (0.6 mL/kg) intraperitoneally twice a week and injected 250 *μ*g EVs obtained from human umbilical cord MSCs in 330 *μ*L PBS directly into the left and right lobes of mouse livers six weeks after the last CCl_4_ treatment [25]. They demonstrated that intrahepatic injection of human umbilical cord MSC-EVs reduced the number of surface fibrous capsules and softened their texture and alleviated hepatic inflammation and collagen deposition in CCl_4_-induced fibrotic liver. Accordingly, our study showed that AMSC-EVs suppressed the fiber accumulation and activation of HSCs. Furthermore, our *in vitro* experiments demonstrated that AMSC-EVs suppressed the HSC activation, which is a crucial event for fibrogenesis [6]. In addition, we demonstrated that AMSC-EVs suppressed the number of KCs that contribute to HSC activation and liver fibrosis [47]. However, AMSC-EVs could not significantly suppress the expression of genes related to liver fibrosis in an *in vivo* model of more severe liver fibrosis, which was achieved by the administration of a higher amount of CCl_4_ for six weeks. Increasing the dose and/or frequency of EV injections might provide a better therapeutic effect.

We previously reported that AMSC transplantation ameliorated CCl_4_-induced liver fibrosis in rats and that the anti-inflammatory effect of AMSC on KCs might contribute to their antifibrotic effect. [14]. Similarly, the present study demonstrated that AMSC-EVs exerted anti-inflammatory and antifibrotic effects. MSCs have been suggested as a novel therapeutic approach for the treatment of inflammatory and fibrotic liver diseases [48]; however, intravascular infusion of MSCs was reported to cause embolism and death in animals, leading to increased interest in non-cell-based therapies rather than cell-based therapies due to the associated ease of manufacturing processes and better safety profile of these nonviable approaches [49–51]. EVs are one of the predominant components associated with paracrine activity, and MSC-derived EVs are desired as a cell-free therapeutic option in regenerative medicine [52]. Furthermore, by replacing MSC transplantation with EVs, many of the associated safety concerns and limitations could be mitigated [53], and MSC-EVs may offer specific advantages for patient safety such as lower propensity to trigger innate and adaptive immune response [54].

## 5. Conclusions

AMSC-EVs ameliorated inflammation and fibrogenesis in rat models of NASH and liver fibrosis, potentially by attenuating HSC and KC activation. AMSC-EV administration should be considered as a new therapeutic strategy for the treatment of chronic liver disease.

## Figures and Tables

**Table 1:** Primer sequences.

Rat TNF-*α* F	GGCTCCCTCTCATCAGTTCCA
Rat TNF-*α* R	CGCTTGGTGGTTTGCTACGA
Rat IL-1*β* F	CCTATGTCTTGCCCGTGGAG
Rat IL-1*β* R	CACACACTAGCAGGTCGTCA
Rat IL-6F	CCCTTCAGGAACAGCTATGAA
Rat IL-6 R	ACAACATCAGTCCCAAGAAGG
Rat MCP-1F	CTGTCTCAGCCAGATGCAGTTAA
Rat MCP-1 R	AGCCGACTCATTGGGATCAT
Rat TGF-*β* F	CTGCTGACCCCCACTGATAC
Rat TGF-*β* R	AGCCCTGTATTCCGTCTCCT
Rat CD68 F	TCACAAAAAGGCTGCCACTCTT
Rat CD68 R	TCGTAGGGCTTGCTGTGCTT
Rat CD11c F	CTGTCATCAGCAGCCACGA
Rat CD11c R	ACTGTCCACACCGTTTCTCC
Rat CD163 F	TGTAGTTCATCATCTTCGGTCC
Rat CD163 R	CACCTACCAAGCGGAGTTGAC
Rat *α*-SMA F	GACACCAGGGAGTGATGGTT
Rat *α*-SMA R	GTTAGCAAGGTCGGATGCTC
Rat collagen1a1 F	GATGGCTGCACGAGTCACAC
Rat collagen1a1 R	ATTGGGATGGAGGGAGTTTA
Rat MMP2 F	CTTGCTGGTGGCCACATTC
Rat MMP2 R	CTCATTCCCTGCGAAGAACAC
Rat TIMP1 F	GACCACCTTATACCAGCGTT
Rat TIMP1 R	GTCACTCTCCAGTTTGCAAG
Rat 18s rRNA F	GCAATTATTCCCCATGAACG
Rat 18s rRNA R	GGCCTCACTAAACCATCCAA
Human TNF-*α* F	CAGCCTCTTCTCCTTCCTGA
Human TNF-*α* R	GCCAGAGGGCTGATTAGAGA
Human *β*-actin F	CCAACCGCGAGAAGATGA
Human *β*-actin R	CCAGAGGCGTACAGGGATAG

F: forward; R: reverse.

## Data Availability

All the data used to support the findings of this study are included within the article.

## Abbreviations

AMSC: Amnion-derived mesenchymal stem cell
CCl_4_: Carbon tetrachloride
CCL: C-C motif chemokine ligand
DMEM: Dulbecco's modified Eagle's medium
EV: Extracellular vesicle
EX: Exosome
FBS: Fetal bovine serum
HFD: High-fat diet
HSA: Human serum albumin
HSC: Hepatic stellate cell
I*κ*B-*α*: Inhibitor *κ*B-*α*
IL: Interleukin
LBP: Lipopolysaccharide-binding protein
LPS: Lipopolysaccharide
MCP-1: Monocyte chemoattractant protein-1
MEM: Minimal essential medium
MSC: Mesenchymal stem cell
MMP: Matrix metalloproteinase
MV: Microvesicle
MyD88: Myeloid differentiation protein 88
NAFLD: Nonalcoholic fatty liver disease
NASH: Nonalcoholic steatohepatitis
NF: Normal skin fibroblast
PBS: Phosphate-buffered saline
SD: Standard deviation
SEM: Scanning electron microscopy
SMA: Smooth muscle actin
TBS: Tris-buffered saline
TGF: Transforming growth factor
TIMP: Tissue inhibitor of metalloproteinases
TNF: Tumor necrosis factor
TRAF: Tumor necrosis factor receptor-associated factor.

## Supplementary Materials

Supplementary Materials Supplementary Figure 1: characterization of NF-EVs. (A) Size distribution of the particles measured with the qNano system. (B) Western blot analysis with anti-CD81 antibody. NF: normal skin fibroblasts; EV: extracellular vesicle. Supplementary Figure 2: effect of AMSC-EVs in rat with HFD-induced steatohepatitis. (A) Expression of CD163. ^∗∗^ *p* < 0.01 versus control. Scale bar, 200 *μ*m. AMSC: amnion-derived mesenchymal stem cell; EV: extracellular vesicle; HFD: high-fat diet.
