# Supplementary material for: Extracellular Vesicles from Amnion-Derived Mesenchymal Stem Cells Ameliorate Hepatic Inflammation and Fibrosis in Rats
Source: Stem Cells Int. 2018 Dec 24;2018:3212643. doi: 10.1155/2018/3212643 (PMC6323530; doi:10.1155/2018/3212643)

Supplementary Material

**Extracellular vesicles from amnion-derived mesenchymal stem cells ameliorate hepatic inflammation and fibrosis in rats**

Masatsugu Ohara, Shunsuke Ohnishi, Hidetaka Hosono, Koji Yamamoto,

Kohei Yuyama, Hideki Nakamura, Qingjie Fu, Osamu Maehara, Goki Suda,

and Naoya Sakamoto

*** Correspondence:** Dr. Shunsuke Ohnishi sonishi@pop.med.hokudai.ac.jp

# Supplementary Data

Figure and Figure legend

Supplementary Figure 1. Characterization of NF-EVs. (A) Size distribution of the particles measured with the qNano system. (B) Western blot analysis with anti-CD81 antibody. NF, normal skin fibroblast; EV, extracellular vesicle.


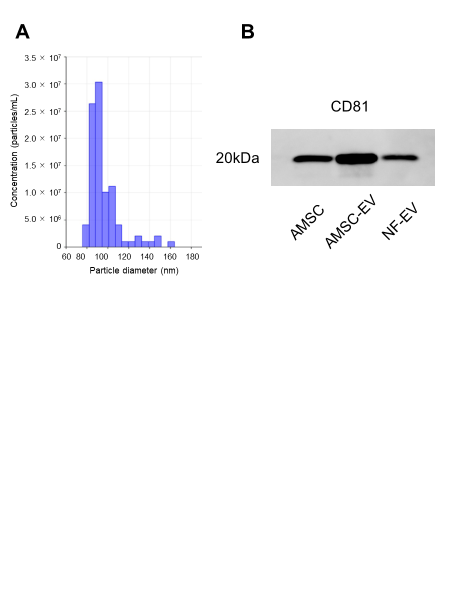


Supplementary Figure 2. Effect of AMSC-EVs in rats with HFD-induced steatohepatitis. (A) Expression of CD163. **p < 0.01 versus control. Scale bar, 200 μm. AMSC, amnion-derived mesenchymal stem cell; EV, extracellular vesicle; HFD, high-fat diet.


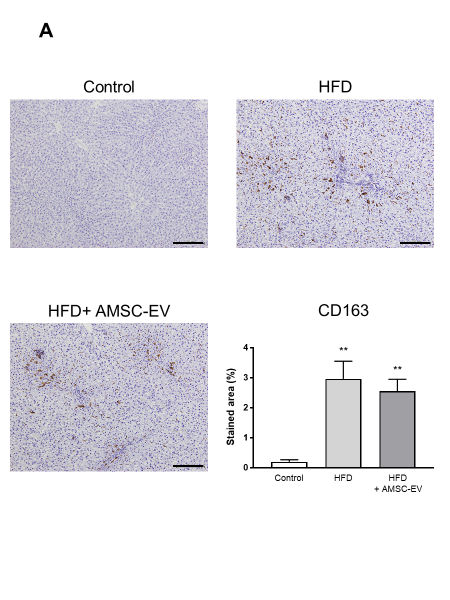

Supplement: Supplementary Materials — Supplementary Figure 1: characterization of NF-EVs. (A) Size distribution of the particles measured with the qNano system. (B) Western blot analysis with anti-CD81 antibody. NF: normal skin fibroblasts; EV: extracellular vesicle. Supplementary Figure 2: effect of AMSC-EVs in rat with HFD-induced steatohepatitis. (A) Expression of CD163. ∗∗ p < 0.01 versus control. Scale bar, 200 μm. AMSC: amnion-derived mesenchymal stem cell; EV: extracellular vesicle; HFD: high-fat diet. [file 3212643.f1.docx]
